# Diaqua­dimethano­lbis[4-(1*H*-tetra­zol-1-yl)benzoato]zinc(II) dihydrate

**DOI:** 10.1107/S1600536808008490

**Published:** 2008-04-04

**Authors:** Shu-Ming Zhang

**Affiliations:** a Department of Chemistry, Nankai University, Tianjin 300071, People’s Republic of China, and School of Chemical Engineering and Technology, Hebei University of Technology, Tianjin 300130, People’s Republic of China

## Abstract

In the title compound, [Zn(C_8_H_5_N_4_O_2_)_2_(CH_3_OH)_2_(H_2_O)_2_]·2H_2_O, the Zn^II^ ion lies on an inversion centre and is coordinated by two O atoms from two 4-(tetra­zol-1-yl)benzoate ligands, two O atoms from two methanol mol­ecules and two O atoms from two water mol­ecules in a slightly distorted octa­hedral geometry. In addition, there are two uncoordinated water mol­ecules in the crystal structure. The crystal structure is stabilized by inter­molecular O—H⋯O hydrogen bonds.

## Related literature

For related literature, see: Zou *et al.* (2005 ▶); Dinca *et al.* (2006 ▶); Li *et al.* (2007 ▶); Zhang & Du (2007 ▶).
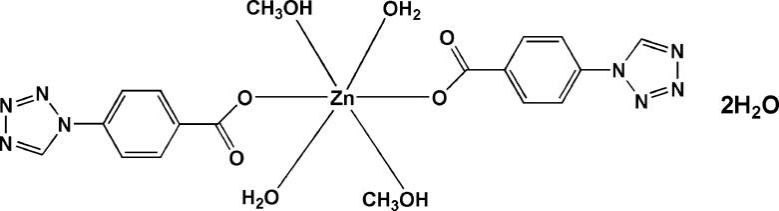


## Experimental

### 

#### Crystal data

[Zn(C_8_H_5_N_4_O_2_)_2_(CH_4_O)_2_(H_2_O)_2_]·2H_2_O
*M* *_r_* = 579.84
Monoclinic, 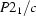

*a* = 13.220 (3) Å
*b* = 7.1551 (14) Å
*c* = 12.636 (3) Å
β = 90.24 (3)°
*V* = 1195.3 (4) Å^3^
*Z* = 2
Mo *K*α radiation
μ = 1.10 mm^−1^
*T* = 293 (2) K
0.20 × 0.18 × 0.16 mm

#### Data collection

Bruker P4 diffractometer
Absorption correction: multi-scan (*SADABS*; Bruker, 1998 ▶) *T*_min_ = 0.810, *T*_max_ = 0.844
12254 measured reflections
2746 independent reflections
2359 reflections with *I* > 2σ(*I*)
*R*_int_ = 0.045

#### Refinement

*R*[*F* ^2^ > 2σ(*F* ^2^)] = 0.032
*wR*(*F* ^2^) = 0.076
*S* = 1.04
2746 reflections
188 parameters
H atoms treated by a mixture of independent and constrained refinement
Δρ_max_ = 0.29 e Å^−3^
Δρ_min_ = −0.23 e Å^−3^

### 

Data collection: *SMART* (Bruker, 1998 ▶); cell refinement: *SAINT* (Bruker, 1998 ▶); data reduction: *SAINT*; program(s) used to solve structure: *SHELXS97* (Sheldrick, 2008 ▶); program(s) used to refine structure: *SHELXL97* (Sheldrick, 2008 ▶); molecular graphics: *SHELXTL* (Sheldrick, 2008 ▶); software used to prepare material for publication: *SHELXTL*.

## Supplementary Material

Crystal structure: contains datablocks I, global. DOI: 10.1107/S1600536808008490/at2552sup1.cif

Structure factors: contains datablocks I. DOI: 10.1107/S1600536808008490/at2552Isup2.hkl

Additional supplementary materials:  crystallographic information; 3D view; checkCIF report

## Figures and Tables

**Table:** 

Zn1—O1	2.0483 (14)
Zn1—O3	2.1078 (15)
Zn1—O1*W*	2.1342 (14)

**Table:** 

O1—Zn1—O3	93.56 (6)
O1—Zn1—O1*W*	91.02 (6)
O3—Zn1—O1*W*	92.25 (6)

**Table 2:** Hydrogen-bond geometry (Å, °)

*D*—H⋯*A*	*D*—H	H⋯*A*	*D*⋯*A*	*D*—H⋯*A*
O1*W*—H1*WA*⋯O2*W*^i^	0.82	1.97	2.759 (2)	160
O2*W*—H2*WB*⋯O1*W*^ii^	0.77 (3)	2.07 (3)	2.831 (2)	175 (3)
O3—H3*M*⋯O2*W*^iii^	0.75 (3)	1.99 (3)	2.726 (2)	167 (3)

Symmetry codes: (i) 
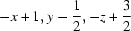
; (ii) 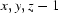; (iii) 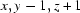.

**Fig. 1.**
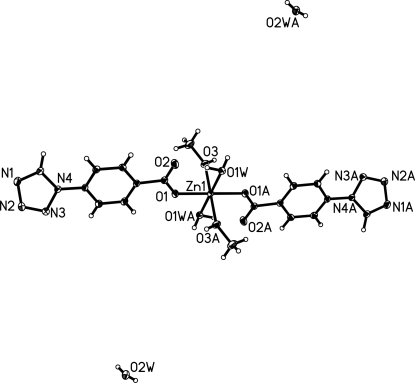
The molecular structure of (I), with 30% probability displacement ellipsoids. [Symmetry code: (A) -x+1, -y, -z+2.]

**Fig. 2.**
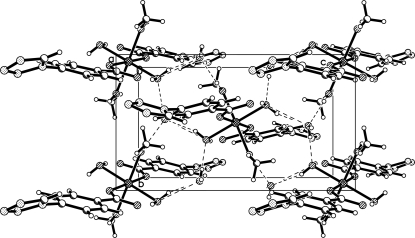
A portion of the crystal stacking structure, showing the intermolecular O—H···O, hydrogen bonds as dashed lines.

## supplementary crystallographic information

### Comment

Coordination architectures formed from 1*H*-tetrazol and its derivatives have attracted wide attentions in recent years, due to not only their fascinating structures and topologies, but also their potential applications in luminescence, magnetism and gas storage (Dinca, *et al.*, 2006; Li, *et al.*, 2007). However, there are rare reports (Zou, *et al.*, 2005) of the coordination systems using the benzoic acids with N-heterocycle as ligands. So we synthesized several coordination compounds by such ligands. And here we report the structure of title compound (I).

The structure of (I) consists of discrete neutral unit [Zn(C_8_H_5_N_4_O_2_)_2_(CH_3_OH)_2_(H_2_O)_2_], and two lattice water molecules (Fig. 1), atom Zn1 lies on an inversion centre and is coordinated by two O atoms from two 4-(tetrazol-1-yl) benzoate ligands, two O atoms from two methanol molecules and two O atoms from two water molecules in a distorted octahedral geometry.The metal ion of (I) is bonded to the carboxyl group of 4-(tetrazol-1-yl) benzoate, which is remarkably different from our previous reported compound that using the same ligand with N donor coordinating to metal ion (Zhang *et al.*, 2007). The crystal stacking of (I) (Fig. 2) is stabilized by the intermolecular O—H···O hydrogen bonds (Table 2).

### Experimental

A solution of Zn(NO_3_)_2_.6H_2_O (0.1 mmol) in water (5 ml) was added to a solution of 4-(tetrazol-1-yl) benzoic acid (38 mg, 0.2 mmol) and sodium hydroxide (8 mg, 0.2 mmol) in methanol (5 mL). The reaction mixture was stirred for 30 min and then filtered. Colourless crystals of (I) suitable for X-ray diffraction were obtained by slow evaporation after two weeks [yield: 46%].

### Refinement

H atoms of C were included in calculated positions and treated in the subsequent refinement as riding atoms, with C—H = 0.93 and 0.96 Å and *U*_iso_(H) = 1.2 and 1.5 *U*_eq_(C,*N*). The H atoms of water was located in Fourier difference map and refined without restraint.

### Crystal data

**Table:**

[Zn(C_8_H_5_N_4_O_2_)_2_(CH_4_O)_2_(H_2_O)_2_]·2H_2_O	*F*_000_ = 600
*M**_r_* = 579.84	*D*_x_ = 1.611 Mg m^−^^3^
Monoclinic, *P*2_1_/*c*	Mo *K*α radiation λ = 0.71073 Å
Hall symbol: -P 2ybc	Cell parameters from 11248 reflections
*a* = 13.220 (3) Å	θ = 3.1–27.6º
*b* = 7.1551 (14) Å	µ = 1.10 mm^−^^1^
*c* = 12.636 (3) Å	*T* = 293 (2) K
β = 90.24 (3)º	Block, colourless
*V* = 1195.3 (4) Å^3^	0.20 × 0.18 × 0.16 mm
*Z* = 2	

### Data collection

**Table:**

Bruker P4 diffractometer	2746 independent reflections
Radiation source: fine-focus sealed tube	2359 reflections with *I* > 2σ(*I*)
Monochromator: graphite	*R*_int_ = 0.045
*T* = 293(2) K	θ_max_ = 27.5º
ω scans	θ_min_ = 3.1º
Absorption correction: multi-scan(SADABS; Bruker, 1998)	*h* = −17→17
*T*_min_ = 0.810, *T*_max_ = 0.844	*k* = −9→9
12254 measured reflections	*l* = −16→16

### Refinement

**Table:**

Refinement on *F*^2^	Hydrogen site location: inferred from neighbouring sites
Least-squares matrix: full	H atoms treated by a mixture of independent and constrained refinement
*R*[*F*^2^ > 2σ(*F*^2^)] = 0.032	*w* = 1/[σ^2^(*F*_o_^2^) + (0.0297*P*)^2^ + 0.5826*P*] where *P* = (*F*_o_^2^ + 2*F*_c_^2^)/3
*wR*(*F*^2^) = 0.076	(Δ/σ)_max_ = 0.001
*S* = 1.04	Δρ_max_ = 0.29 e Å^−^^3^
2746 reflections	Δρ_min_ = −0.23 e Å^−^^3^
188 parameters	Extinction correction: SHELXL97 (Sheldrick, 2008), Fc^*^=kFc[1+0.001xFc^2^λ^3^/sin(2θ)]^-1/4^
Primary atom site location: structure-invariant direct methods	Extinction coefficient: 0.0228 (12)
Secondary atom site location: difference Fourier map	

### Special details

**Table:**

Geometry. All e.s.d.'s (except the e.s.d. in the dihedral angle between two l.s. planes) are estimated using the full covariance matrix. The cell e.s.d.'s are taken into account individually in the estimation of e.s.d.'s in distances, angles and torsion angles; correlations between e.s.d.'s in cell parameters are only used when they are defined by crystal symmetry. An approximate (isotropic) treatment of cell e.s.d.'s is used for estimating e.s.d.'s involving l.s. planes.
Refinement. Refinement of *F*^2^ against ALL reflections. The weighted *R*-factor *wR* and goodness of fit *S* are based on *F*^2^, conventional *R*-factors *R* are based on *F*, with *F* set to zero for negative *F*^2^. The threshold expression of *F*^2^ > σ(*F*^2^) is used only for calculating *R*-factors(gt) *etc*. and is not relevant to the choice of reflections for refinement. *R*-factors based on *F*^2^ are statistically about twice as large as those based on *F*, and *R*- factors based on ALL data will be even larger.

### Fractional atomic coordinates and isotropic or equivalent isotropic displacement parameters (Å^2^)

**Table:**

	*x*	*y*	*z*	*U*_iso_*/*U*_eq_	
Zn1	0.5000	0.0000	1.0000	0.02298 (11)	
O1	0.63696 (9)	0.0452 (2)	0.92928 (10)	0.0313 (3)	
O2	0.73211 (10)	0.1468 (2)	1.06367 (10)	0.0399 (4)	
C2	0.97854 (12)	0.0991 (2)	0.77039 (14)	0.0231 (4)	
N4	1.06694 (11)	0.0940 (2)	0.70586 (12)	0.0243 (3)	
C5	0.80990 (13)	0.1028 (2)	0.89760 (14)	0.0228 (4)	
N3	1.06465 (12)	0.1453 (3)	0.60316 (13)	0.0339 (4)	
C4	0.90418 (13)	0.1480 (3)	0.93867 (14)	0.0259 (4)	
H4	0.9104	0.1793	1.0098	0.031*	
N1	1.21854 (12)	0.0669 (3)	0.64661 (14)	0.0342 (4)	
C7	0.88492 (14)	0.0580 (3)	0.72624 (15)	0.0282 (4)	
H7	0.8786	0.0294	0.6547	0.034*	
C3	0.98863 (14)	0.1470 (3)	0.87560 (15)	0.0274 (4)	
H3	1.0516	0.1782	0.9035	0.033*	
C8	0.71954 (13)	0.0989 (3)	0.96960 (14)	0.0256 (4)	
C1	1.16184 (14)	0.0462 (3)	0.72993 (16)	0.0291 (4)	
H1	1.1840	0.0047	0.7958	0.035*	
C6	0.80137 (14)	0.0608 (3)	0.79125 (15)	0.0279 (4)	
H6	0.7380	0.0339	0.7629	0.033*	
N2	1.15560 (13)	0.1290 (3)	0.56869 (14)	0.0375 (4)	
O3	0.54347 (11)	−0.2616 (2)	1.06193 (13)	0.0380 (4)	
C9	0.63908 (16)	−0.3252 (3)	1.0949 (2)	0.0470 (6)	
H9A	0.6907	−0.2579	1.0578	0.071*	
H9B	0.6451	−0.4563	1.0799	0.071*	
H9C	0.6467	−0.3050	1.1696	0.071*	
O2W	0.43377 (14)	0.4734 (2)	0.16558 (13)	0.0340 (3)	
O1W	0.54904 (11)	0.14327 (19)	1.13919 (10)	0.0289 (3)	
H1WA	0.5471	0.0718	1.1898	0.043*	
H2WA	0.378 (2)	0.457 (4)	0.156 (2)	0.053 (9)*	
H2WB	0.463 (2)	0.382 (4)	0.156 (2)	0.061 (10)*	
H1WB	0.612 (2)	0.155 (4)	1.121 (2)	0.058 (8)*	
H3M	0.5060 (19)	−0.325 (4)	1.088 (2)	0.047 (8)*	

### Atomic displacement parameters (Å^2^)

**Table:**

	*U*^11^	*U*^22^	*U*^33^	*U*^12^	*U*^13^	*U*^23^
Zn1	0.01705 (16)	0.02848 (18)	0.02340 (17)	0.00002 (12)	−0.00056 (11)	0.00133 (13)
O1	0.0173 (6)	0.0487 (9)	0.0279 (7)	−0.0049 (6)	0.0003 (5)	0.0000 (6)
O2	0.0265 (7)	0.0670 (11)	0.0261 (7)	−0.0095 (7)	0.0019 (6)	−0.0061 (7)
C2	0.0174 (9)	0.0241 (9)	0.0279 (9)	−0.0001 (7)	0.0021 (7)	0.0030 (7)
N4	0.0191 (7)	0.0275 (8)	0.0261 (8)	0.0008 (6)	−0.0003 (6)	0.0027 (7)
C5	0.0184 (8)	0.0231 (9)	0.0270 (9)	−0.0014 (7)	0.0000 (7)	0.0016 (7)
N3	0.0277 (9)	0.0455 (10)	0.0284 (9)	0.0043 (7)	0.0021 (7)	0.0072 (8)
C4	0.0240 (9)	0.0310 (10)	0.0227 (9)	−0.0040 (7)	−0.0023 (7)	0.0004 (8)
N1	0.0239 (9)	0.0384 (9)	0.0403 (10)	0.0029 (7)	0.0040 (7)	0.0031 (8)
C7	0.0244 (9)	0.0352 (10)	0.0249 (9)	−0.0018 (8)	−0.0025 (8)	−0.0039 (8)
C3	0.0190 (9)	0.0330 (10)	0.0301 (10)	−0.0040 (7)	−0.0053 (7)	0.0009 (8)
C8	0.0199 (9)	0.0283 (10)	0.0287 (10)	−0.0013 (7)	−0.0004 (7)	0.0038 (8)
C1	0.0214 (9)	0.0331 (11)	0.0328 (10)	0.0023 (7)	−0.0020 (8)	0.0010 (8)
C6	0.0179 (9)	0.0362 (10)	0.0294 (10)	−0.0033 (7)	−0.0039 (7)	−0.0030 (8)
N2	0.0290 (9)	0.0478 (11)	0.0358 (10)	0.0035 (8)	0.0080 (7)	0.0093 (8)
O3	0.0261 (7)	0.0373 (9)	0.0505 (9)	0.0028 (7)	−0.0007 (7)	0.0194 (7)
C9	0.0323 (12)	0.0468 (14)	0.0620 (15)	0.0093 (10)	−0.0041 (11)	0.0120 (12)
O2W	0.0277 (8)	0.0361 (9)	0.0382 (8)	−0.0020 (7)	0.0031 (7)	−0.0057 (7)
O1W	0.0251 (7)	0.0359 (8)	0.0257 (7)	0.0002 (6)	0.0004 (6)	0.0029 (6)

### Geometric parameters (Å, °)

**Table:**

Zn1—O1	2.0483 (14)	C4—H4	0.9300
Zn1—O1^i^	2.0483 (14)	N1—C1	1.304 (3)
Zn1—O3	2.1078 (15)	N1—N2	1.361 (2)
Zn1—O3^i^	2.1078 (15)	C7—C6	1.379 (3)
Zn1—O1W^i^	2.1342 (14)	C7—H7	0.9300
Zn1—O1W	2.1342 (14)	C3—H3	0.9300
O1—C8	1.263 (2)	C1—H1	0.9300
O2—C8	1.247 (2)	C6—H6	0.9300
C2—C3	1.379 (3)	O3—C9	1.405 (2)
C2—C7	1.387 (2)	O3—H3M	0.75 (3)
C2—N4	1.428 (2)	C9—H9A	0.9600
N4—C1	1.334 (2)	C9—H9B	0.9600
N4—N3	1.349 (2)	C9—H9C	0.9600
C5—C6	1.381 (3)	O2W—H2WA	0.75 (3)
C5—C4	1.386 (2)	O2W—H2WB	0.77 (3)
C5—C8	1.505 (2)	O1W—H1WA	0.8200
N3—N2	1.286 (2)	O1W—H1WB	0.87 (3)
C4—C3	1.375 (3)		
			
O1—Zn1—O1^i^	180.00 (3)	C6—C7—C2	118.22 (17)
O1—Zn1—O3	93.56 (6)	C6—C7—H7	120.9
O1^i^—Zn1—O3	86.44 (6)	C2—C7—H7	120.9
O1—Zn1—O3^i^	86.44 (6)	C4—C3—C2	119.00 (17)
O1^i^—Zn1—O3^i^	93.56 (6)	C4—C3—H3	120.5
O3—Zn1—O3^i^	180.0	C2—C3—H3	120.5
O1—Zn1—O1W^i^	88.98 (6)	O2—C8—O1	125.41 (17)
O1^i^—Zn1—O1W^i^	91.02 (6)	O2—C8—C5	117.92 (16)
O3—Zn1—O1W^i^	87.75 (6)	O1—C8—C5	116.66 (16)
O3^i^—Zn1—O1W^i^	92.25 (6)	N1—C1—N4	109.28 (17)
O1—Zn1—O1W	91.02 (6)	N1—C1—H1	125.4
O1^i^—Zn1—O1W	88.98 (6)	N4—C1—H1	125.4
O3—Zn1—O1W	92.25 (6)	C7—C6—C5	121.35 (17)
O3^i^—Zn1—O1W	87.75 (6)	C7—C6—H6	119.3
O1W^i^—Zn1—O1W	180.0	C5—C6—H6	119.3
C8—O1—Zn1	129.52 (12)	N3—N2—N1	110.75 (16)
C3—C2—C7	121.54 (17)	C9—O3—Zn1	129.90 (14)
C3—C2—N4	118.74 (16)	C9—O3—H3M	105 (2)
C7—C2—N4	119.72 (16)	Zn1—O3—H3M	122 (2)
C1—N4—N3	107.84 (15)	O3—C9—H9A	109.5
C1—N4—C2	130.34 (16)	O3—C9—H9B	109.5
N3—N4—C2	121.81 (15)	H9A—C9—H9B	109.5
C6—C5—C4	118.98 (17)	O3—C9—H9C	109.5
C6—C5—C8	121.50 (16)	H9A—C9—H9C	109.5
C4—C5—C8	119.52 (16)	H9B—C9—H9C	109.5
N2—N3—N4	106.50 (15)	H2WA—O2W—H2WB	109 (3)
C3—C4—C5	120.86 (17)	Zn1—O1W—H1WA	109.5
C3—C4—H4	119.6	Zn1—O1W—H1WB	96.5 (17)
C5—C4—H4	119.6	H1WA—O1W—H1WB	107.7
C1—N1—N2	105.63 (15)		
			
O1^i^—Zn1—O1—C8	73 (100)	Zn1—O1—C8—C5	174.81 (12)
O3—Zn1—O1—C8	−78.56 (17)	C6—C5—C8—O2	−176.77 (19)
O3^i^—Zn1—O1—C8	101.44 (17)	C4—C5—C8—O2	3.7 (3)
O1W^i^—Zn1—O1—C8	−166.24 (17)	C6—C5—C8—O1	3.9 (3)
O1W—Zn1—O1—C8	13.76 (17)	C4—C5—C8—O1	−175.56 (17)
C3—C2—N4—C1	33.9 (3)	N2—N1—C1—N4	−0.2 (2)
C7—C2—N4—C1	−146.7 (2)	N3—N4—C1—N1	0.4 (2)
C3—C2—N4—N3	−144.73 (19)	C2—N4—C1—N1	−178.39 (18)
C7—C2—N4—N3	34.7 (3)	C2—C7—C6—C5	−0.2 (3)
C1—N4—N3—N2	−0.4 (2)	C4—C5—C6—C7	1.8 (3)
C2—N4—N3—N2	178.53 (17)	C8—C5—C6—C7	−177.68 (18)
C6—C5—C4—C3	−1.5 (3)	N4—N3—N2—N1	0.2 (2)
C8—C5—C4—C3	177.97 (17)	C1—N1—N2—N3	0.0 (2)
C3—C2—C7—C6	−1.8 (3)	O1—Zn1—O3—C9	31.19 (19)
N4—C2—C7—C6	178.77 (17)	O1^i^—Zn1—O3—C9	−148.81 (19)
C5—C4—C3—C2	−0.4 (3)	O3^i^—Zn1—O3—C9	−165 (73)
C7—C2—C3—C4	2.1 (3)	O1W^i^—Zn1—O3—C9	120.04 (19)
N4—C2—C3—C4	−178.49 (17)	O1W—Zn1—O3—C9	−59.96 (19)
Zn1—O1—C8—O2	−4.4 (3)		

Symmetry codes: (i) −*x*+1, −*y*, −*z*+2.

### Hydrogen-bond geometry (Å, °)

**Table:**

*D*—H···*A*	*D*—H	H···*A*	*D*···*A*	*D*—H···*A*
O1W—H1WA···O2W^ii^	0.82	1.97	2.759 (2)	160
O2W—H2WB···O1W^iii^	0.77 (3)	2.07 (3)	2.831 (2)	175 (3)
O3—H3M···O2W^iv^	0.75 (3)	1.99 (3)	2.726 (2)	167 (3)

Symmetry codes: (ii) −*x*+1, *y*−1/2, −*z*+3/2; (iii) *x*, *y*, *z*−1; (iv) *x*, *y*−1, *z*+1.
